# Impaired autophagy in amyloid-beta pathology: A traditional review of recent Alzheimer's research

**DOI:** 10.7555/JBR.36.20220145

**Published:** 2022-09-28

**Authors:** Minghao Yuan, Yangyang Wang, Zhenting Huang, Feng Jing, Peifeng Qiao, Qian Zou, Jing Li, Zhiyou Cai

**Affiliations:** 1 Chongqing Medical University, Chongqing 400042, China; 2 Department of Neurology, Chongqing General Hospital, University of Chinese Academy of Sciences, Chongqing 400013, China; 3 Department of Neurology, Chongqing School, University of Chinese Academy of Sciences, Chongqing 400013, China; 4 Chongqing Key Laboratory of Neurodegenerative Diseases, Chongqing 400013, China

**Keywords:** Alzheimer's disease, autophagy, amyloid-beta, amyloid precursor protein secretases, metabolism

## Abstract

Alzheimer's disease (AD) is the most prevalent neurodegenerative disorder. The major pathological changes in AD progression are the generation and accumulation of amyloid-beta (Aβ) peptides as well as the presence of abnormally hyperphosphorylated tau proteins in the brain. Autophagy is a conserved degradation pathway that eliminates abnormal protein aggregates and damaged organelles. Previous studies have suggested that autophagy plays a key role in the production and clearance of Aβ peptides to maintain a steady-state of Aβ peptides levels. However, a growing body of evidence suggests that autophagy is significantly impaired in the pathogenesis of AD, especially in Aβ metabolism. Therefore, this article reviews the latest studies concerning the mechanisms of autophagy, the metabolism of Aβ peptides, and the defective autophagy in the production and clearance of Aβ peptides. Here, we also summarize the established and new strategies for targeting autophagy *in vivo* and through clinical AD trials to identify gaps in our knowledge and to generate further questions.

## Introduction

Alzheimer's disease (AD) is an age-related neurodegenerative disease that is characterized by extensive memory loss, ongoing cognitive dysfunction, and behavioural disorders^[1–3]^. Global cost of AD is estimated to be 818 billion US dollars, which puts a huge strain on political economy^[4]^. The number of people diagnosed with AD is also expected to rise from 46.8 million in 2015 to 74.7 million by 2030. This trajectory suggests that this figure will rise to 131.5 million by 2050^[4]^, if we do not develop effective interventions. Unfortunately, AD begins to develop more than 10 years before symptoms begin to manifest^[5]^. Research to date has suggested that various alterations, including the production and accumulation of amyloid-beta (Aβ) and phosphorylated tau proteins, as well as mitochondrial and synaptic damage, inflammatory responses, oxidative stress, hormonal imbalance, and neuronal loss, are involved in the AD progression^[6–14]^. These pathological changes are persistent in Aβ peptide aggregation that in turn forms senile plaques and drives the accumulation of hyperphosphorylated tau proteins. This creates neurofibrillary tangles that in turn impairs nervous system function^[1,15–19]^ and accelerates deterioration. Therefore, understanding these mechanisms could provide insights into the development and progression of AD as well as possible therapeutic targets.

Autophagy is a self-cannibalisation process that involves the decomposition of cell structures through lysosomes, maintaining a balance in both synthesis, degradation, and cellular circulation^[20–22]^. Autophagy can be both selective or non-selective, depending on the properties of specific target proteins^[23]^. In most cases, autophagy refers to macroautophagy that is an evolutionarily conserved catabolic process involved in vesicle formations, *i.e.*, autophagosomes, which engulf cellular macromolecules and organelles. The tightly coordinated multi-step process involved in autophagy is associated with protein products from autophagy-associated genes, which adhere to the target and participate in the entire cycle^[24]^. Under normal circumstances, autophagy plays a type of "housekeeping" role in cellular homeostasis by removing denatured or misfolded proteins and damaged organelles as we age. Induced by various stress signals including certain nutrients, hypoxia, and reactive oxygen species^[13,25]^, soluble proteins and organelles in the cytoplasm are degraded into amino acids to supply energy and for biosynthesis.

Aβ has also been found to have a continuously toxic effect on neurons and synaptic facilitation^[16]^. However, while impaired autophagy has previously been identified in AD^[26–29]^, only recently has autophagy been found to mediate Aβ secretion into extracellular space^[27]^. In an AD brain, autophagosomes and late autophagic vacuoles accumulate in dystrophic neurites, suggesting that there is underlying autophagy impairment^[27,29]^. Additionally, the selective accumulation of lysosomal dense bodies in the brains of AD cases implies that there is abnormal autolysosomal proteolysis^[30–31]^. However, the precise alterations in autophagy processes in AD remain unclear.

This review highlights impaired autophagy in the pathogenesis of AD. We initially describe Aβ metabolization, the pathway of autophagy, and the role of autophagy in neurodegenerative diseases. This is followed by a comprehensive summary of defective autophagy in promoting an Aβ precursor protein (APP) processing that leads to an enhanced activity or increased levels of the beta-site amyloid precursor protein cleaving enzyme 1 (BACE1) and γ-secretase. This again accelerates the production of Aβ into a state of overproduction. We then discuss the association between defective autophagy and Aβ clearance failures. In addition, we provide a summary of compounds and drugs that target autophagy in AD animal models or AD patients.

## Autophagy

**Figure 1:**
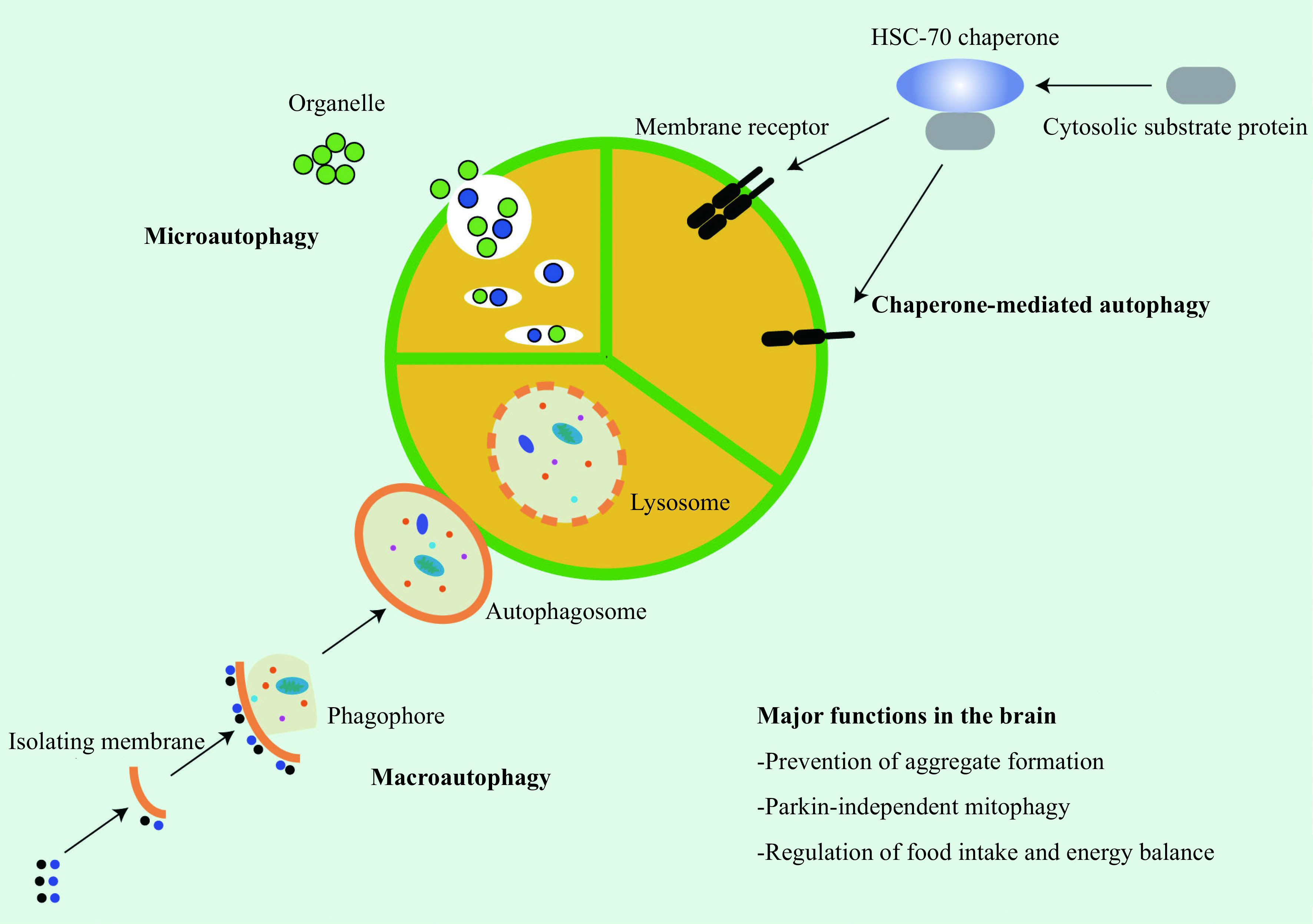
An overview of autophagy.

Autophagy is a natural self-degradation process that promotes cell survival in response to nutritional stress that can be caused by starvation and by certain diseases. Autophagy also balances sources of cellular building materials and energy intake. The literal meaning of autophagy is '*self-eating*', as 'auto' means *self* and 'phagy' means *to* *eat*. During this aptly named "self-eating" process, cells remove unwanted molecules and dysfunctional cellular components. The process is divided into macroautophagy, microautophagy and chaperone-mediated autophagy according to the mechanism through which intracellular materials are delivered into lysosomes for degradation^[20]^. The different types of autophagy are illustrated in ***Fig. 1*** and described in detail below.

Macroautophagy starts with the formation of a double-membraned vesicles called autophagosomes^[32–33]^, and then cytoplasmic components, such as organelles and proteins, are enveloped by double-membrane vesicles^[33]^. The outer membrane of the autophagosome then fuses with lysosomes to form autolysosomes, where organelles and proteins are degraded by lysosomal acid hydrolase. In microautophagy, lysosomes directly degrade cytoplasmic components that are engulfed by invagination of the membrane^[20]^. It is generally accepted that microautophagy can be either non-selective or selective^[32]^. Selective autophagy includes mitophagy, pexophagy, reticulophagy, ribophagy, lipophagy, xenophagy, nucleophagy and ferritinophagy^[34]^, which can target specific tissues, malignant cells, damaged organelles, aggregated proteins, invasive pathogens, and excessive peroxisomes^[35]^. It is achieved by the autophagy receptor that adheres to the target *via* the autophagy-related protein 8 (ATG8) family on the autophagosome membrane. Then poly-ubiquitin on the target is recognized by receptor proteins, which initiates selective autophagy. In chaperone-mediated autophagy, cytoplasmic proteins are degraded in lysosomes or vacuoles after being transported by molecular chaperones, such as heat shock cognate protein 70^[23]^ (***Fig. 1***).

**Figure 2:**
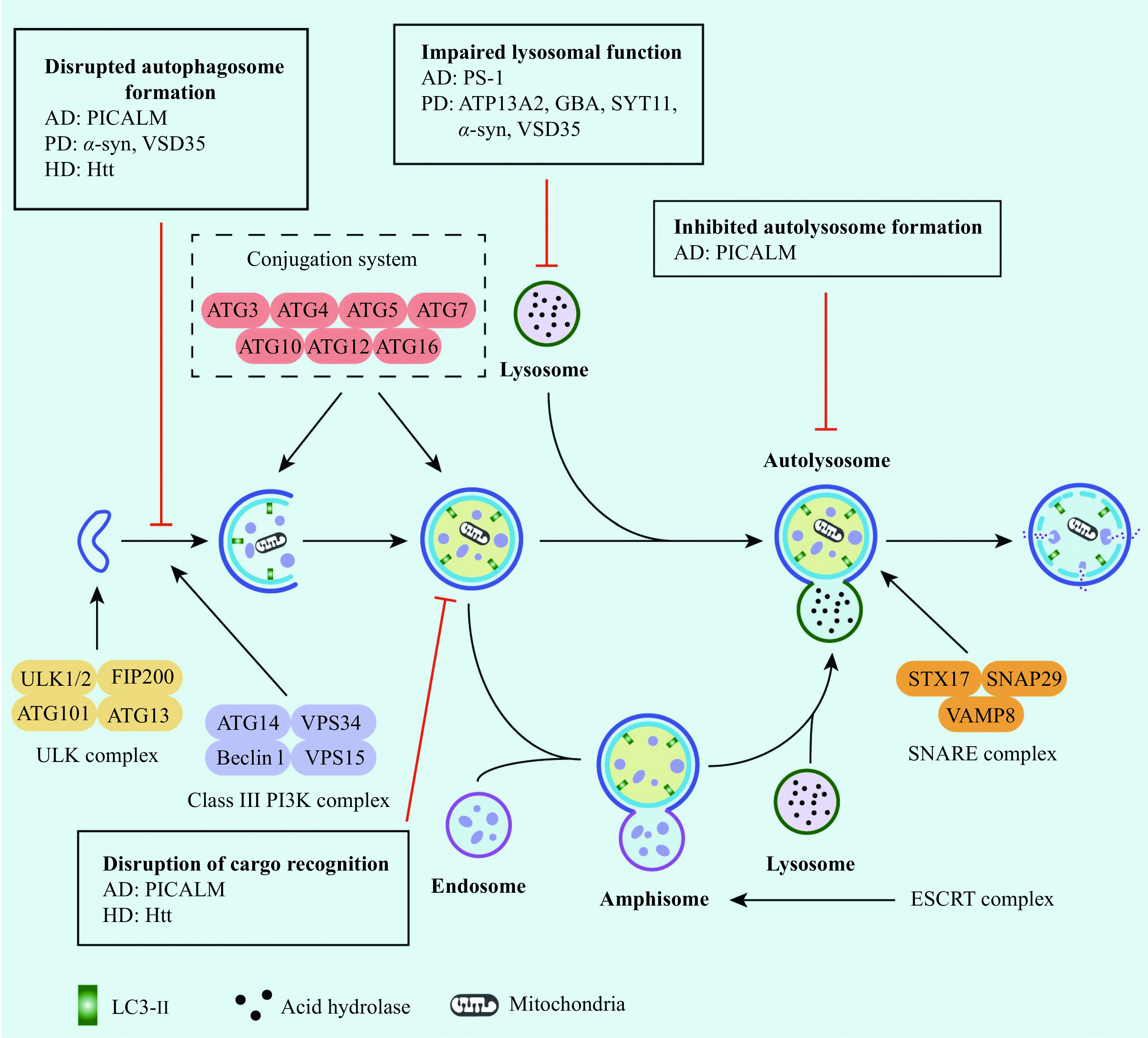
The general processes involved in autophagy and impaired autophagy in neurodegenerative disorders.

In summary, autophagy is a highly conserved pathway that degrades large proteins and organelles by lysosomes (***Fig. 1***), which plays a vital role in the circulation, infection, immunity, and metabolism of cells^[36–37]^. Under normal developmental conditions, cells perform low levels of autophagy, specifically basal autophagy, to maintain cellular homoeostasis. However, under various stressors, autophagy becomes highly dynamic, and its progress can be split into initiation, nucleation, elongation, closure, and lysosomal fusion^[38–41]^ (***Fig. 2***).

### Initiation

The activity of the unc-51-like kinase 1 (ULK1) complex (comprising ULK1/ATG1, ATG13, FAK family kinase-interacting protein of 200 kDa, and ATG101) is required for the initiation of autophagy^[42]^. The ULK1 complex is affected by certain stress signaling pathways, such as the mammalian target of the rapamycin (mTOR) pathway^[43]^.

### Nucleation

Nucleation of a double-membrane structure called the phagophore requires a class Ⅲ phosphatidylinositol 3-kinase complex. This complex consists of vacuolar protein sorting 34 (VPS34), VPS15, ATG14, and Beclin1 and generates phosphatidylinositol-3 phosphate, which is essential for nucleation of vesicles^[44]^.

### Elongation and closure

The elongation and closure required to form autophagosomes depend on two ubiquitin-like conjugation systems, known as the ATG12-ATG5 system and the ATG8/LC3-lipid phosphatidylethanolamine (PE) system^[45–47]^. These systems interact and regulate each other with the participation of ubiquitin activases (E1) and ubiquitin conjugases (E2). ATG12, activated by ATG7, combines with ATG5 through transport of ATG10 and then binds to ATG16 to form a multi-body complex of ATG12-ATG5-ATG16^[44]^. This complex is located on the surface of the outer membrane of the vacuole and participates in the elongation of this membrane. LC3-Ⅰ, which is mediated by ATG7 and ATG3, conjugates with PE to form LC3-Ⅱ to participate in membrane elongation^[45]^. LC3-Ⅱ, commonly regarded as a marker of autophagy, is also an important signaling regulatory protein located on the membrane of autophagic vacuoles.

### Lysosomal fusion

A fusion of lysosomes with autophagosomes to form an autolysosome is the final step in autophagy^[45]^. As specialized organelles, lysosomes play an important role in breaking down extracellular materials and recycling cellular components in different pathways^[48]^. The fusion between autophagosomes and lysosomes requires a soluble N-ethylmaleimide-sensitive factor activating the protein receptor complex that comprises syntaxin 17, synaptosomal-associated protein 29, and vesicle-associated membrane protein 8^[49]^.

### Formation of amphisomes

An autophagosome can also fuse with a late endosome (LE) to form an amphisome that contains markers of both autophagosomes (LC3) and endosomes (ras-related protein in brain 5 [RAB5], RAB7, and RAB11)^[50]^. Members of the endosomal sorting complex required for complex transport play key roles in formation of amphisomes and autolysosomes during autophagy^[51]^.

### Degradation and reuse

The inner membranes and components of autolysosomes are degraded by lysosomal hydrolases, and a series of lysosomal proteases (*e.g.*, cathepsins B, D, and L) degrade the contents in mammalian cells. When macromolecules have been degraded in lysosomes, monomer molecules, such as amino acids and lipids, are recycled into the cytoplasm for reuse; however, little is known about what happens in this stage. Atg22 is essential for the reuse in yeast, but no counterparts of Atg22 have been found in mammals^[52]^. Additionally, little is known about the role of autophagy in the reuse of large molecules, such as carbohydrates and lipids.

## Amyloid-beta metabolism

The two hallmark pathological changes, which are required for a diagnosis of AD, are deposition of the extracellular Aβ plaque and neurofibrillary tangles comprised of microtubule-binding protein tau. Following a decade of research, the excessive production and impaired Aβ clearance are now known to cause these amyloid plaques and deleterious cascades involved in the pathogenesis of AD.

### Amyloid-beta production

**Figure 3:**
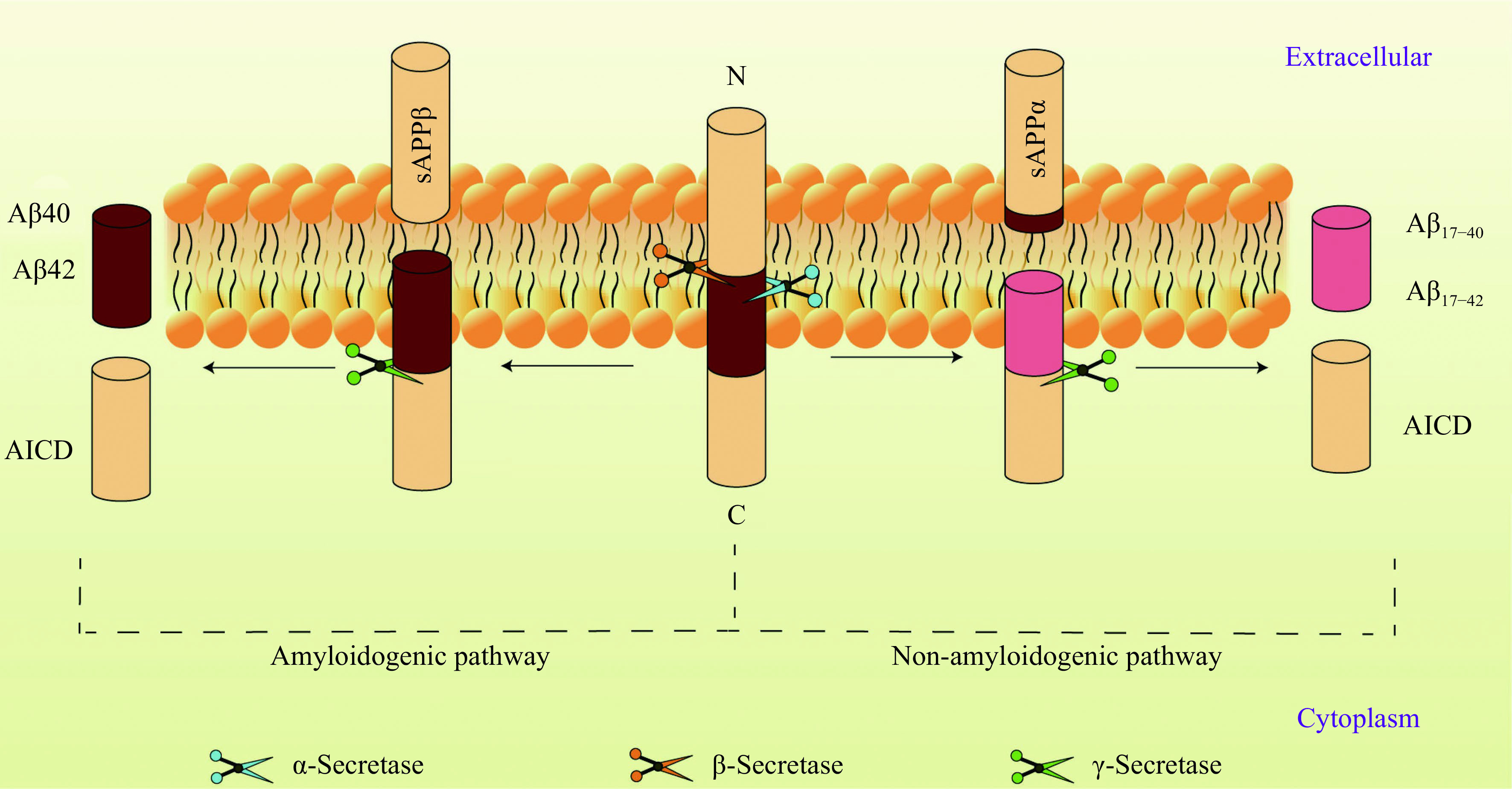
Amyloid-beta precursor protein processing.

Aβ is produced by way of sequential cleavage of the APP by β- and γ-secretases^[53]^. APP, which is a type Ⅰ transmembrane protein, exists in various tissues and is intensively expressed on the membrane of neuronal synapses^[54]^. There are two main metabolic pathways for APP: a non-amyloidogenic and an amyloidogenic pathways (***Fig. 3***). The non-amyloidogenic pathway is mediated by α- and γ-secretases, soluble Aβ precursor protein-alpha (sAPPα), p3 peptides (Aβ_17–40_ and Aβ_17–42_), and the APP intracellular domain, but not toxic Aβ peptides, are produced, because the cutting site of α-secretase is inside the Aβ sequence^[55–56]^. Whereas, in the amyloidogenic pathway, Aβ is produced and released by a combination of BACE1 and γ-secretase in the intracellular compartments, including the trans-Golgi network, endosomes, and autophagosomes^[57]^. Aβ40 and Aβ42 are the most common peptides of Aβ subtypes. In comparison with Aβ40, Aβ42 is more hydrophobic and prone to aggregation, forming oligomers or fibrils and finally senile plaques, which is one of the main histological features of AD^[57–60]^. However, there is no evidence of an association between the number of senile plaques and the severity or progression of AD^[57]^.

Due to different roles the of α-, β-, and γ-secretases in the metabolism of APP, the products they produce, when cleaving APP, have different cellular effects. The α-secretase enzyme plays a major role in the production of sAPPα. Upregulated α-secretase enzyme can promote the production of sAPPα and reduce production of Aβ, whereas the β-secretase acts as a rate-limiting enzyme in the formation of Aβ. The γ-secretase enzyme plays a pivotal role in catalysing the production of Aβ in the metabolism of APP and in determining the terminal stage of Aβ. In general, upregulation of α-secretase activity and inhibition of β- and γ-secretases are important for reducing Aβ formation.

### Amyloid-beta clearance

In the brain, neprilysin and insulin-degrading enzyme are considered to be the main enzymes involved in Aβ degradation^[61]^. Neprilysin, a type Ⅱ metalloproteinase, is responsible for extracellular degradation of Aβ42, and its activation can reduce the aggregation and toxicity related to Aβ, leading potentially to cognitive improvements^[58,62]^. The insulin-degrading enzyme is a mercaptan metalloproteinase with an important role in degrading soluble Aβ monomer and reducing the deposition and accumulation of Aβ^[58,60]^. Additionally, M2 microglia and astrocytes can clear Aβ through phagocytosis. Apolipoprotein E and low-density lipoprotein receptor-related protein 1 (LRP-1) have been demonstrated to assist astrocytes with degradation^[56,60]^. Moreover, LRP-1 can mediate the transport of Aβ across the blood-brain barrier (BBB) to the peripheral blood circulation^[56,63–64]^, and brain microvascular endothelial cells at the BBB participate in the clearance of Aβ *via* LRP-1 and P-glycoprotein^[63,65]^.

### Amyloid-beta in Alzheimer's disease

For some time, it has been assumed that an imbalance between the production and clearance of Aβ leads to its accumulation in the brain. Normally, a dynamic balance is maintained between the generation and decomposition of Aβ in the brain. However, when various pathogenic factors are present, the Aβ metabolism is disrupted, causing its accumulation that affects neurological function^[66]^. In the early stage of AD, extensive aggregation and accumulation of Aβ may occur in the brain, leading to pathological changes, including impaired autophagy^[67]^, apoptosis^[68]^, and oxidative stress^[69]^ as well as abnormal growth of axons^[70]^.

In addition, abnormal accumulation of Aβ is known to be an initial factor in the inflammatory response observed in AD. Senile plaques formed by Aβ promote an inflammatory response, causing proliferation of glia^[71]^. Accumulated Aβ binds to the receptor on microglial cell membranes and facilitates secretion of inflammatory factors from microglia, inducing an inflammatory response, whereas activated inflammatory factors impair normal neurons nearby^[72–74]^. Furthermore, a recent study has indicated that microglia activated by Aβ plaques promotes the propagation Aβ into unaffected brain regions^[75]^. It is therefore reasonable to suggest that the Aβ pathology may interact with pathological changes in the brain, causing a vicious circle that eventually accelerates the progression of AD.

## Impaired autophagy induces over-generation of amyloid-beta

Aβ peptides are generated from APP through sequential cleavage by BACE1 and the γ-secretase complex^[54,56]^. A recent study has indicated that pathological Rab5 overactivation during AD induces endosomal dysfunction, which disrupts the formation of autolysosomes, eventually causing autophagy dysfunction^[76]^. Additionally, a detective autophagy, characterized by a deficit in autolysosome acidification in neurons, has been found to occur before extracellular amyloid deposition in five AD mice models^[77]^. Given the importance of autophagy in Aβ metabolism, it is possible that an impaired autophagy enhances activities or levels of the β-secretase and γ-secretase, resulting in Aβ overproduction.

### Impaired autophagy and BACE1 in Alzheimer's disease

BACE1 is a key enzyme in the amyloidogenic pathway of APP and is responsible for regulating the degradation of APP to produce Aβ. BACE1 is a transmembrane aspartic protease that is highly expressed in brain neurons. There is a compelling evidence for an age-associated increased activity of BACE1 in the AD brain^[78]^. As a result, much research has focused on BACE1 as a target for the treatment and prevention of AD^[79–81]^. BACE1 is initially synthesized into a protein precursor in the endoplasmic reticulum, before being transported to the Golgi body, where it is glycosylated and modified into a mature BACE1 protein. Mature BACE1 is then internalized through the plasma membrane or transferred from the trans-Golgi network directly to the endosome, providing a suitable acidic environment for the protein's activity^[82–84]^. Eventually, BACE1 is degraded within the lysosomes.

Previous studies have indicated that autophagosomes are constantly produced in the distal axons and move retrogradely to the soma for lysosomal proteolysis^[85–89]^. This transport is triggered by fusion of the nascent autophagosome with LE and driven by dynein-SNAPIN complexes with LE^[88]^. Interestingly, autophagosomes have been shown to transport BACE1, thereby regulating its trafficking and degradation^[90]^. Both *in vivo* and *in vitro* studies have shown that a large amount of BACE1 is recruited into autophagy vesicles and transported to the soma, augmenting the transport of BACE1 to lysosomes for degradation^[90]^. Therefore, autophagy is considered another pathway for trafficking of BACE1 for degradation in neurons. However, under pathological conditions, an AD-associated defective autophagy causes an impaired retrograde transport, which results in an accumulation of BACE1, enhancing the processing of APP by BACE1 in axons^[91]^. Increased BACE1 in distal axons augments production of Aβ in the APP process, exacerbating the AD pathological changes. Therefore, a potential therapeutic strategy that increases the induction of autophagy to improve the trafficking of BACE1 may be reasonable for treating the early stage of AD in the future.

In addition to regulating the trafficking and degradation of BACE1, the disruptions of upstream pathways, including phosphatidylinositol 3-kinase/Akt/mTOR and PPARγ/AMPK/mTOR, can indirectly decrease BACE1 levels by activating autophagy. mTORC1, which is a serine-threonine protein kinase, is a classical regulatory molecule for autophagy. This protein kinase inhibits autophagy by binding to the ULK1 complex and phosphorylating both ATG13 and ULK1^[43]^. However, an aberrant up-regulation of the mTOR signaling was detected in AD brains^[29,92–94]^, suggesting an enhanced mTOR activity and an inhibited autophagy during the AD process^[95]^. Otherwise, several recent studies have shown that inhibition of the mTOR pathway can reduce BACE1 levels, decreasing production of Aβ^[96–97]^. These findings suggest that an elevated mTOR activity inhibits autophagy in AD, resulting in increased BACE1 levels and subsequently Aβ over-generation.

### Impaired autophagy causes overactivation of the γ-secretase

The protein γ-secretase complex includes presenilin, nicastrin, anterior pharynx defective-1, and presenilin enhancer 2, which plays a key role in the catabolic metabolization of APP. In cell experiments, an autophagy inhibitor (3-methyladenine) has been found to activate certain components of the γ-secretase complex and significantly increase extracellular levels of Aβ42^[98]^. Furthermore, in AD patients, a defective autophagy can upregulate the expression of these components, thereby increasing the activity of the γ-secretase and promoting the Aβ production. Ohta K *et al* demonstrated that an impaired autophagy stimulated expression of PS1 and activated the γ-secretase^[28]^. When the autophagy-lysosomal system is impaired, the intracellular supply of amino acids decreases, because autophagy is required to maintain amino acid levels. Cellular amino acid deficiencies can lead to elevations in uncharged tRNA levels, which directly activate the environmental sensing protein, the general control non-derepressible 2(GCN2). As part of the amino acid imbalance, an increased GCN2 level causes phosphorylation of eIF2α that regulates the activating transcription factor 4. Finally, presenilin-1 is upregulated, and the γ-secretase is activated, leading to production of Aβ^[28]^.

## Impaired autophagy affects amyloid-beta clearance

Numerous studies have shown a potential association among autophagy, and Aβ clearance and deposition^[27,99–105]^. Large amounts of Aβ deposition interfere with the function of intracellular organelles, such as lysosomes, thereby increasing the accumulation of Aβ and promoting AD progression^[104]^. Aβ-derived diffusible ligands (ADDLs) can significantly reduce phosphorylated p70S6K expression, suggesting that the mTOR pathway inhibits an abnormal autophagy involved in an ADDL-induced autophagy^[106]^. Moreover, levels of Beclin-1, a key protein for initiation of autophagy, is significantly decreased in the early stage of AD, indicating that a reduced Beclin-1 level promotes neurodegeneration and accelerates the accumulation of Aβ^[102–103]^. Furthermore, autophagy also participates in the secretion of Aβ *via* the secretory pathway from the endoplasmic reticulum to the Golgi body and then to the plasma membrane or secretory lysosomal pathway^[27,107]^. Defective autophagy has been found to decrease the intracellular Aβ peptide load and accumulation of the extracellular Aβ in mice, which suggests that abnormal autophagy can reduce the degradation of Aβ, but also indicates that autophagy plays a role in secretion of Aβ^[27]^. Further studies on the dual roles of autophagy in the clearance and secretion of Aβ may contribute to a better understanding of the pathogenesis of AD.

Several recent studies have demonstrated that enhancement or activation of autophagy increases Aβ clearance and reduces its deposition. For example, Caccamo *et al* demonstrated that an upregulation of p62 expression in an AD mouse model *via* the mTOR-dependent pathway, which activates autophagy, reduced level of Aβ and cognitive defects^[99]^. By contrast, Wang *et al*^[108]^ found that the ratio of LC3B-Ⅱ/LC3B-Ⅰ increased in AD mice that received an oral rAAV/Aβ vaccination, and autophagy was enhanced with a decreased p62 level. However, the role of p62 in mediating autophagy to clear Aβ remains controversial and requires further investigation.

Rapamycin has been found to prevent an increase in calcium ions and to decrease the mitochondrial membrane potential in PC12 cells^[109]^. These alterations indicate that a moderate activation of autophagy can regulate a dynamic balance of calcium ions and maintain the stability of mitochondrial membrane potential, thereby alleviating the cytotoxicity induced by Aβ^[109]^. Additionally, mouse modelling research performed by Di Meco *et al* showed that after 12 weeks treatment with 12/15-lipoxygenase inhibitors, the level of Aβ was significantly decreased, and the effect was dependent on activation of autophagy in neurons^[100]^.

In conjunction with these findings, investigators have also found that recovery of the autophagic flux can actually reverse the manifestations of AD caused by an accumulation of Aβ. The zinc ion carrier effect of hydroxychloroquine increases autophagic flux and reduces the accumulation of Aβ^[105]^. The epidermal growth factor receptor ErbB2 is thought to be dormant in the adult brain, but is activated in the hippocampus in AD patients. ErbB2 can dissociate Beclin-1 from the Vps34-Vps15 complex to inhibit the autophagic flux, suggesting that upregulated ErbB2 causes defective autophagy in AD^[105]^. When ErbB2 was downregulated in an AD mouse study, spatial learning and cognitive function appeared to be significantly improved, indicating that the downregulated ErbB2 can reverse inhibition of the autophagic flux and enhance the clearance of Aβ^[110]^.

**Figure 4:**
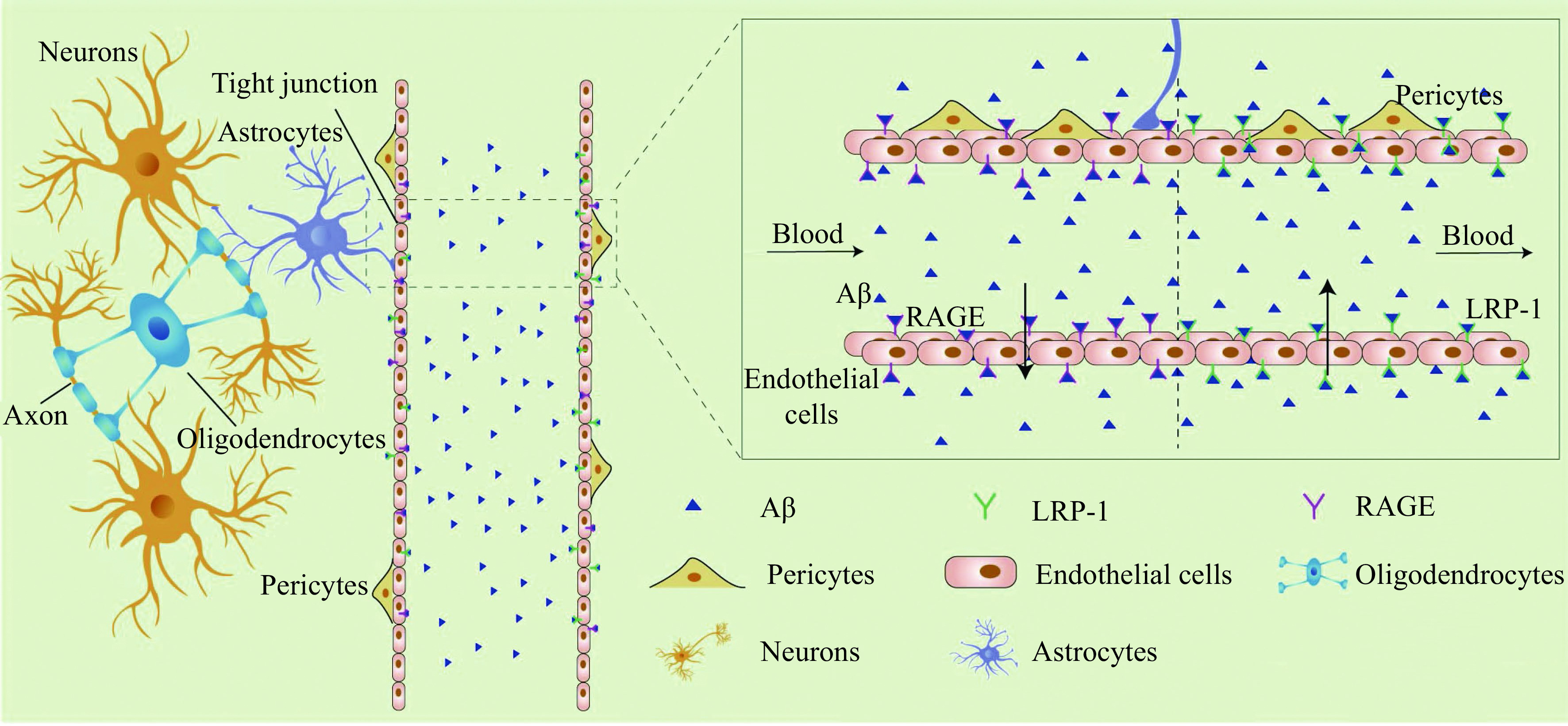
Amyloid-beta transport at the BBB is regulated by RAGE and LRP-1.

Generally, the intracellular domain of LRP-1 binds to phosphatidylinositol-binding clathrin assembly protein to regulate endosomal transcytosis of Aβ at the BBB^[111–112]^ (***Fig. 4***). However, cerebral accumulation of Aβ has been shown to accelerate autophagy-lysosomal degradation of LRP-1 in endothelial cells at the BBB, leading to dysfunction at the BBB^[101]^. Unlike LRP-1, the receptor for advanced glycation end products (RAGE) transports Aβ from the systemic circulation into the brain. RAGE is thought to be an important factor mediating the cytotoxicity of Aβ and promoting AD pathogenesis. Moreover, RAGE has been reported to impair autophagy-lysosomal degradation, indirectly disrupting Aβ clearance^[108]^. Further investigation has revealed that the Aβ oligomer causes impaired tight junction proteins *via* RAGE-mediated autophagy^[113]^. However, the precise relationship among autophagy, LRP-1, and RAGE in AD is not clear and requires further investigation.

## Established and new strategies targeting autophagy in Alzheimer's disease

As has been previously discussed, Aβ and tau pathology serve as key roles in AD pathogenesis. At present, numerous pre-clinical studies or clinical trials focus on inducing or enhancing autophagy in AD treatments^[114–118]^. Activation of autophagy reduces Aβ and tau accumulation in AD animal models, which alleviates AD-related impairment in brains. Major regulations targeting different molecules lead to AMPK activation, mTORC1 inhibition, and transcription factor EB (TFEB) activation, which directly or indirectly enhance autophagy^[19]^. Therefore, we highlight and summarize the potential compounds and drugs that potentially play neuroprotective roles by enhancing autophagy *in vivo* or in clinical trials in ***Table 1*** and ***Table 2***.

**Table 1:** Autophagy enhancers in Alzheimer's disease animal models

Compound	Mechanism	Target	Model	Reference
Metformin	AMPK activation	AMPK	SAMP8 mice APP/PS1 mice Tg6799 mice	[114] [120] [142]
Trehalose	AMPK activation	SLC2A	APP/PS1 mice	[143]
Nilotinib	AMPK activation	c-ABL inhibitor	Tg-APP mice	[144]
Resveratrol	AMPK activation	SIRT1	APP/PS1 mice	[121]
Berberine	AMPK activation	unknown	3×Tg mice	[125,145]
Rapamycin	mTORC1 inhibition	FKBP12	PDAPP mice 3×Tg-AD mice	[127,146] [128,147]
Everolimus	mTORC1 inhibition	FKBP12	3×Tg mice	[129]
Temsirolimus	mTORC1 inhibition	mTOR	APP/PS1 mice Tg30 mice	[130] [148]
Latrepirdine	mTORC1 inhibition	unknown	TgCRND8 mice	[141]
Carbamazepine	mTORC1 inhibition	Na^+^ channel inhibitor	APP/PS1 mice	[149]
Rifampicin	mTORC1 inhibition	PP2A	Tg2576 mice	[150]
Curcumin analogue C1	TFEB activation	TFEB	5×FAD mice 3×Tg mice	[117]
HEP14	TFEB activation	PKC	APP/PS1 mice	[135]
Aspirin	TFEB activation	PPARα	5×FAD mice	[151]
Gemfibrozil and Wy14643	TFEB activation	PPARα	APP-PSEN1ΔE9 mice	[115]
Cinnamic acid	TFEB activation	PPARα	5×FAD mice	[152]
Gypenoside XVII	TFEB activation	unknown	APP/PS1 mice	[153]
Lithium	cAMP/IP3	IMP	APP/PS1 mice	[154]
AMPK: adenosine 5'-monophosphate (AMP)-activated protein kinase; cAMP: cyclic Adenosine monophosphate; FKBP12: FK506-binding protein 12; IMP: inosine monphosphate; mTOR: mammalian target of rapamycin; PKC: protein kinase C; PP2A: protein phosphatase 2A; PPARα: peroxisome proliferators-activated receptor α; SLC2A: solute carrier 2A; SIRT1: silent mating type information regulation 2 homolog- 1; TFEB: transcription factor EB.				

**Table 2:** Autophagy enhancers in clinical trials of Alzheimer's disease

Drug	ClinicalTrials.gov identifier	Phase	Enrollment (*N*)	Status	Result	Reference
Metformin	NCT01965756	2	20	Completed	Metformin benefited cognition symptomatically and modified disease pathology with high safety and good tolerance.	[155]
	NCT04098666	2/3	370	Recruiting	N/A	[156]
	NCT00620191	2	80	Completed	After adjusting for baseline Alzheimer's Disease Assessment Scale-cognitive subscale, metformin improved the total recall of the selective reminding test.	[137]
Rapamycin	NCT04629495	2	40	Recruiting	N/A	[157]
	NCT04200911	1	10	Active	N/A	[158]
Lithium	NCT01055392	2	61	Unknown	Lithium-treated patients remained stable on cognition over two years and with a significant increase in CSF Aβ42 after 3 years.	[138,159–160]
	NCT03185208	4	80	Active	N/A	[161]
	NCT02129348	2	77	Completed	Lithium is safe for outcomes. In exploratory analyses, the improvement on lithium was greater than placebo on neuropsychiatric inventory delusions and irritability/lability.	[139–140]
	NCT00088387	2	35	Completed	N/A	[162]
Latrepirdine	NCT00912288	3	86	Terminated	This study was terminated due to the lack of demonstration of efficacy in the completed DIM14 CONNECTION (B1451002/NCT00675623) study.	[163]
	NCT00377715	2	183	Completed	Benefits in ADAS-cog compared with control.	[164]
	NCT00838110	3	742	Completed	Latrepirdine did not significantly improve ADAS-cog and CIBIC-plus.	[165]
Resveratrol	NCT00678431	3	739	Completed	Low-dose resveratrol is safe and well tolerated; however, the role of resveratrol in AD is not clear.	[166]
	NCT01504854	2	119	Completed	Resveratrol reduced CSF MMP9 and Aβ levels, but not MAPT levels. Resveratrol attenuated declines in mini-mental status examination scores.	[167–168]
Nilotinib	NCT02947893	2	42	Unknown	N/A	[169]
Trehalose	NCT04663854	1	20	Recruiting	N/A	[170]
N/A: Not yet published; Aβ: amyloid-beta; AD: Alzheimer's disease; CSF: cerebrospinal fluid; MAPT: microtubule-associated protein tau; MMP9: matrix metalloprotein 9.						

### Autophagy enhancers in pre-clinical animal models

Various small molecules, such as metformin, resveratrol, nilotinib and berberine, activate AMPK to induce autophagy, which plays neuroprotective roles in AD animal models^[119]^. Metformin is an anti-diabetic drug that can activate AMPK, and improve cognitive dysfunction *via* reducing Aβ plaque loading and phosphorylated tau levels in AD models^[114,120]^; however, the role of Metformin in AD is controversial due to the lack of in-depth studies. Resveratrol, as a natural polyphenol widely distributed in edible food, can activate AMPK and bind to SIRT1 to induce autophagy. Autophagy-dependent and -independent effects of resveratrol on the Aβ metabolism were successively reported to explore the precise mechanism of resveratrol in AD^[121–122]^. Berberine is an AMPK activator that has multiple biological activities, including metabolic anti-diabetes and anti-hypercholesterolemia^[123–124]^. Berberine has been found to induce autophagy and improve learning and memory function by promoting Aβ degradation in AD animal models^[125]^. These findings imply that multiple AMPK-dependent autophagy enhancers exert neuroprotective effects in AD animal models.

Apart from AMPK activators, inhibiting mTOR is another important way to induce autophagy. Rapamycin, as a classical inhibitor of mTORC1, directly induces autophagy combating neurodegenerative disorders, such as AD. Rapamycin can promote autophagy through binding to the cytosolic protein FKBP1A/FKBP12 (FK-binding protein 12)^[126]^. Furthermore, reduced levels of Aβ and rescued cognitive decline were detected after rapamycin treatment in 3XTg and hAPP(J20) mice^[127–128]^. However, the role of rapamycin in AD may be partly involved in other pathways, since rapamycin is a non-autophagy-specific compound. Given the side effects, such as glucose intolerance and hyperlipidemia caused by chronic rapamycin treatment, specific mTORC1 inhibitors, including Everolimus and Temsirolimus, have been developed. Everolimus has been found to inhibit autophagy, reduce Aβ levels and ameliorate cognitive deficits in AD mice^[129]^. In addition, inhibited autophagy and reduced levels of Aβ were widely found in the brain of APP/PS1 mice treated with Temsirolimus^[130]^. Other compounds, such as latrepirdine, carbamazepine and rifampicin, may be also promising anti-AD candidates. However, future studies on the underlying mechanisms of these compounds in AD are highly anticipated.

TFEB is an important transcription factor regulating cell function. Activated TFEB induces the expression of multiple autophagy-related genes^[131]^, which promotes Aβ degradation and attenuates AD development through the autophagy-lysosomal pathway^[132–133]^. As previously mentioned, the autophagy-lysosomal pathway is impaired during AD progression. Therefore, upregulation of TFEB may a promising therapeutic strategy for AD targeting Aβ degradation through enhancing autophagy-lysosomal pathway^[133]^. Curcumin analogue C1 has also been shown to activate TFEB-mediated autophagy-lysosomal biogenesis, and further promote Aβ clearance in 5XFAD and 3XTg mice^[117,134]^. In addition to C1, HEP14 (5β-O-angelate-20-deoxyingenol) has been found to promote TFEB activation through binding to and activating PKCα and PKCδ^[135]^. This HEP14-mediated TFEB activation in the brain of APP/PS1 mice enhances the clearance of Aβ in these mice^[135]^. Other small molecule TFEB activators, including aspirin, cinnamic acid, gemfibrozil and gypenoside XVII, were associated with a decreased Aβ pathological changes in AD animal modelling. Overall, these findings provide a broad evidence for an emerging AD treatment strategy.

### Autophagy enhancers in clinical trials of Alzheimer's disease

To date, multiple autophagy activators have been presented in clinical trials to test for their efficacy in AD patients. Current studies have reported that Metformin is safe and well-tolerated, improving cognitive deficiencies in AD patients^[136–137]^. Additionally, a relatively small phase Ⅱ/Ⅲ trial including 370 patients has been tested to investigate the effects of Metformin on AD progression (NCT04098666). Although, rapamycin has been widely reported in autophagy activation and neuroprotection under various AD animal models, an investigation on its safety and feasibility for AD patients has only just begun (NCT04629495). By contrast, lithium has been well-studied through clinical AD trials. MCI patients who were treated with lithium for two years did not seem to have a significant cognitive decline according to the Alzheimer's Disease Assessment Scale-Cognitive Subscale (ADAS-Cog) and the Clinical Dementia Rating scale (CDR-SoB) scores^[138]^. Additionally, a significant increase in CSF Aβ contents was detected in the lithium treatment group^[138]^, which suggests that lithium has a neuroprotective role in AD patients.

Similarly, positive effects of lithium on behaviours of the AD patients have been demonstrated in recent studies^[139–140]^. Although latrepirdine had been found to alleviate neuropathic Aβ *via* restoring autophagy impairment* in vivo*^[141]^, clinical trials showed that this drug did not improve cognitive dysfunction, and two ongoing studies were terminated (NCT00912288 and NCT00838110). These differences in efficacy between animal models and AD patients may infer that latrepirdine has other functions unrelated to autophagy. However, other drugs, such as resveratrol, nilotinib and trehalose, have provided anti-AD effects according to different results. Collectively, clinical trials suggest that autophagy enhancers may be emerging therapeutic strategies for AD patients. However, given that autophagy enhancers have a wide variety of targets, large-scale studies of autophagic markers are still required.

## Conclusions and perspectives

Autophagy is a necessary process involved in removing proteins prone to aggregation and neuronal intracytoplasmic deposition, both of which result in neurodegenerative disease. Autophagy is closely related to AD pathogenesis, and defective autophagy, which includes the accumulation of autophagic vesicles, decreased autophagic flux, failure of autophagosome maturation and defective autolysosome formation, which have been observed in AD. Impaired autophagy increases levels of Aβ production and reduces Aβ clearance, also it causes degradation failures in BACE1 and APP and activates the γ-secretase to promote Aβ production and accumulation. Correlation between defective autophagy and Aβ clearance failure has now been confirmed. Therefore, targeting autophagic regulation may provide a viable new therapeutic approach for treating AD. The findings of multiple studies suggest various potential therapeutic targets for autophagy, but their individual efficacies are yet to be investigated.

## References

[1] (2012). Alzheimer mechanisms and therapeutic strategies. Cell.

[2] (2020). Characterization of a new molecule capable of inhibiting several steps of the amyloid cascade in Alzheimer's disease. Neurobiol Dis.

[3] (2020). Mitochondrial dysfunction: a potential therapeutic target to treat alzheimer's disease. Mol Neurobiol.

[b4] 4Alzheimer's Disease Intermational. World alzheimer report 2015: the global impact of dementia: an analysis of prevalence, incidence, cost and trends[R]. London: Alzheimer's Disease Intermational, 2015.

[5] (2004). Early Aβ accumulation and progressive synaptic loss, gliosis, and tangle formation in AD brain. Neurology.

[6] (2007). Intracellular amyloid-β in Alzheimer's disease. Nat Rev Neurosci.

[7] (2006). Mitochondrial oxidative damage in aging and Alzheimer's disease: implications for mitochondrially targeted antioxidant therapeutics. J Biomed Biotechnol.

[8] (2012). Abnormal mitochondrial dynamics and synaptic degeneration as early events in Alzheimer's disease: implications to mitochondria-targeted antioxidant therapeutics. Biochim Biophys Acta Mol Basis Dis.

[9] (2010). Cellular stress responses, the hormesis paradigm, and vitagenes: novel targets for therapeutic intervention in neurodegenerative disorders. Antioxid Redox Signaling.

[10] (2009). Vitagenes, cellular stress response, and acetylcarnitine: relevance to hormesis. BioFactors.

[11] (2012). Hormesis: why it is important to biogerontologists. Biogerontology.

[12] (2011). HSF1-dependent upregulation of Hsp70 by sulfhydryl-reactive inducers of the KEAP1/NRF2/ARE pathway. Chem Biol.

[13] (2003). Elevation of mitochondrial glutathione by γ-glutamylcysteine ethyl ester protects mitochondria against peroxynitrite-induced oxidative stress. J Neurosci Res.

[14] (2006). Bilirubin: an endogenous scavenger of nitric oxide and reactive nitrogen species. Redox Rep.

[15] (2012). National Institute on Aging-Alzheimer's Association guidelines for the neuropathologic assessment of Alzheimer's disease. Alzheimer's Dement.

[16] (2018). Amyloid toxicity in Alzheimer's disease. Rev Neurosci.

[17] (2001). Alzheimer's disease: genes, proteins, and therapy. Physiol Rev.

[18] (2019). ERβ promotes Aβ degradation *via* the modulation of autophagy. Cell Death Dis.

[19] (2022). Impairment of the autophagy-lysosomal pathway in Alzheimer's diseases: pathogenic mechanisms and therapeutic potential. Acta Pharm Sin B.

[20] (2010). Autophagy: cellular and molecular mechanisms. J Pathol.

[21] (2021). Guidelines for the use and interpretation of assays for monitoring autophagy (4th edition). Autophagy.

[22] (2022). Lysosome lipid signalling from the periphery to neurons regulates longevity. Nat Cell Biol.

[23] (2019). Amyloid beta and phosphorylated Tau-induced defective autophagy and mitophagy in alzheimer's disease. Cells.

[24] (2013). Mitochondrial degradation during starvation is selective and temporally distinct from bulk autophagy in yeast. FEBS Lett.

[25] (2010). Towards the global understanding of the autophagy regulatory network. Autophagy.

[26] (2009). A central role for autophagy in Alzheimer-type neurodegeneration. Autophagy.

[27] (2014). Dual roles for autophagy: degradation and secretion of Alzheimer's disease Aβ peptide. BioEssays.

[28] (2010). Autophagy impairment stimulates PS1 expression and γ-secretase activity. Autophagy.

[29] (2019). Autophagic dysfunction in Alzheimer's disease: Cellular and molecular mechanistic approaches to halt Alzheimer's pathogenesis. J Cell Physiol.

[30] (2018). Altered γ-secretase processing of APP disrupts lysosome and autophagosome function in monogenic Alzheimer's disease. Cell Rep.

[31] (2017). Amyloid precursor protein and endosomal-lysosomal dysfunction in Alzheimer's disease: inseparable partners in a multifactorial disease. FASEB J.

[32] (2007). Selective and non-selective autophagic degradation of mitochondria in yeast. Autophagy.

[33] (2000). BNIP3 and genetic control of necrosis-like cell death through the mitochondrial permeability transition pore. Mol Cell Biol.

[34] (2016). Ubiquitin-dependent and independent signals in selective autophagy. Trends Cell Biol.

[35] (2016). Mechanisms of selective autophagy. J Mol Biol.

[36] (2015). Autophagy as a pro-death pathway. Immunol Cell Biol.

[37] (2015). Eaten alive: novel insights into autophagy from multicellular model systems. Trends Cell Biol.

[38] (2010). Autophagy and the integrated stress response. Mol Cell.

[39] (2016). Mammalian autophagy: how does it work?. Annu Rev Biochem.

[40] (2017). Molecular definitions of autophagy and related processes. EMBO J.

[41] (2018). Autophagy pathway: cellular and molecular mechanisms. Autophagy.

[42] (2013). The autophagosome: origins unknown, biogenesis complex. Nat Rev Mol Cell Biol.

[43] (2016). Structure and function of the ULK1 complex in autophagy. Curr Opin Cell Biol.

[44] (2018). Using *Drosophila* models of amyloid toxicity to study autophagy in the pathogenesis of Alzheimer's disease. BioMed Res Int.

[45] (2017). Emerging mechanisms in initiating and terminating autophagy. Trends Biochem Sci.

[46] (2011). Autophagy: renovation of cells and tissues. Cell.

[47] (2013). Two ubiquitin-like conjugation systems that mediate membrane formation during autophagy. Essays Biochem.

[48] (2016). The lysosome as a regulatory hub. Annu Rev Cell Dev Biol.

[49] (2012). The hairpin-type tail-anchored SNARE syntaxin 17 targets to autophagosomes for fusion with endosomes/lysosomes. Cell.

[50] (2008). Induction of autophagy promotes fusion of multivesicular bodies with autophagic vacuoles in k562 cells. Traffic.

[51] (2018). ESCRT and autophagies: endosomal functions and beyond. Semin Cell Dev Biol.

[52] (2007). Autophagy: process and function. Genes Dev.

[53] (2013). Phosphatidylinositol-3-phosphate regulates sorting and processing of amyloid precursor protein through the endosomal system. Nat Commun.

[54] (1995). Processing of the beta-amyloid precursor protein and its regulation in Alzheimer's disease. J Neurochem.

[55] (2012). Early and selective impairments in axonal transport kinetics of synaptic cargoes induced by soluble amyloid β-protein oligomers. Traffic.

[56] (2013). Neuropsychological correlates of capacity determinations in Alzheimer disease: implications for assessment. Am J Geriatr Psychiatry.

[57] (2018). Are N- and C-terminally truncated Aβ species key pathological triggers in Alzheimer's disease?. J Biol Chem.

[58] (2013). Neprilysin and Aβ clearance: impact of the APP intracellular domain in NEP regulation and implications in Alzheimer's disease. Front Aging Neurosci.

[59] (2014). The low-density lipoprotein receptor-related protein 1 and amyloid-β clearance in Alzheimer's disease. Front Aging Neurosci.

[60] (2014). ApoE and Aβ in Alzheimer's disease: accidental encounters or partners?. Neuron.

[61] (2006). Clearance of amyloid-beta in Alzheimer's disease: progress, problems and perspectives. Drug Discov Today.

[62] (2016). Differential roles of M1 and M2 microglia in neurodegenerative diseases. Mol Neurobiol.

[63] (2000). Clearance of Alzheimer's amyloid-β_1-40_ peptide from brain by LDL receptor-related protein-1 at the blood-brain barrier. J Clini Invest.

[64] (2015). Physiological amyloid-beta clearance in the periphery and its therapeutic potential for Alzheimer's disease. Acta Neuropathol.

[65] (2018). Ganglioside-mediated assembly of amyloid β-protein: roles in Alzheimer's disease. Prog Mol Biol Transl Sci.

[66] (2012). LRP1 in brain vascular smooth muscle cells mediates local clearance of Alzheimer's amyloid-β. J Neurosci.

[67] (2005). Extensive involvement of autophagy in Alzheimer disease: an immuno-electron microscopy study. J Neuropathol.

[68] (2022). Trauma-like exposure alters neuronal apoptosis, Bin1, Fkbp5 and NR2B expression in an amyloid-beta (1–42) rat model of Alzheimer's disease. Neurobiol Learn Mem.

[69] (2019). Oxidative stress, dysfunctional glucose metabolism and Alzheimer disease. Nat Rev Neurosci.

[70] (2022). Walnut oil reduces Aβ levels and increases neurite length in a cellular model of early Alzheimer disease. Nutrients.

[71] (2012). The role of astrocytes in amyloid β-protein toxicity and clearance. Exp Neurol.

[72] (2014). Microglia, neuroinflammation, and beta-amyloid protein in Alzheimer's disease. Int J Neurosci.

[73] (2019). Neuropathological correlates and genetic architecture of microglial activation in elderly human brain. Nat Commun.

[74] (2015). Differential regulation of resolution in inflammation induced by amyloid-β42 and lipopolysaccharides in human microglia. J Alzheimers Dis.

[75] (2022). Microglia contribute to the propagation of Aβ into unaffected brain tissue. Nat Neurosci.

[76] (2020). Endosomal dysfunction induced by directly overactivating Rab5 recapitulates prodromal and neurodegenerative features of Alzheimer's disease. Cell Rep.

[77] (2022). Faulty autolysosome acidification in Alzheimer's disease mouse models induces autophagic build-up of Aβ in neurons, yielding senile plaques. Nat Neurosci.

[78] (2004). β-secretase activity increases with aging in human, monkey, and mouse brain. Am J Pathol.

[79] (2009). The β-secretase enzyme BACE in health and Alzheimer's disease: regulation, cell biology, function, and therapeutic potential. J Neurosci.

[80] (2009). Function, regulation and therapeutic properties of β-secretase (BACE1). Semin Cell Dev Biol.

[81] (2010). BACE: therapeutic target and potential biomarker for Alzheimer's disease. Int J Biochem Cell Biol.

[82] (2000). Maturation and endosomal targeting of β-site amyloid precursor protein-cleaving enzyme: the Alzheimer's disease β-secretase. J Biol Chem.

[83] (2003). Elevated β-secretase expression and enzymatic activity detected in sporadic Alzheimer disease. Nat Med.

[84] (2012). BACE1 protein endocytosis and trafficking are differentially regulated by ubiquitination at lysine 501 and the Di-leucine motif in the carboxyl terminus. J Biol Chem.

[85] (2011). Lysosomal proteolysis inhibition selectively disrupts axonal transport of degradative organelles and causes an Alzheimer's-like axonal dystrophy. J Neurosci.

[86] (2012). Autophagosome assembly and cargo capture in the distal axon. Autophagy.

[87] (2014). Autophagosome biogenesis in primary neurons follows an ordered and spatially regulated pathway. Dev Cell.

[88] (2015). Axonal autophagosomes recruit dynein for retrograde transport through fusion with late endosomes. J Cell Biol.

[89] (2016). Compartment-specific regulation of autophagy in primary neurons. J Neurosci.

[90] (2017). Autophagy-mediated Regulation of BACE1 protein trafficking and degradation. J Biol Chem.

[91] (2016). Molecular genetics of early-onset Alzheimer's disease revisited. Alzheimers Dement.

[92] (2019). Rapamycin and Alzheimer's disease: time for a clinical trial?. Sci Transl Med.

[93] (2015). The mammalian target of rapamycin at the crossroad between cognitive aging and Alzheimer's disease. NPJ Aging Mech Dis.

[94] (2015). Alteration of mTOR signaling occurs early in the progression of Alzheimer disease (AD): analysis of brain from subjects with pre-clinical AD, amnestic mild cognitive impairment and late-stage AD. J Neurochem.

[95] (2022). Protein degradation-associated mechanisms that are affected in Alzheimer´s disease. Mol Cell Biochem.

[96] (2015). Lamotrigine reduces β-site AβPP-cleaving enzyme 1 protein levels through induction of autophagy. J Alzheimer's Dis.

[97] (2018). Dihydroceramide desaturase 1 inhibitors reduce amyloid-β levels in primary neurons from an alzheimer's disease transgenic model. Pharm Res.

[98] (2015). Autophagy dysfunction upregulates beta-amyloid peptides *via* enhancing the activity of γ-secretase complex. Neuropsychiatr Dis Treat.

[99] (2017). p62 improves AD-like pathology by increasing autophagy. Mol Psychiatry.

[100] (2017). 12/15-Lipoxygenase inhibition reverses cognitive impairment, brain amyloidosis, and tau pathology by stimulating autophagy in aged triple transgenic mice. Biol Psychiatry.

[101] (2019). Amyloid-beta impairs insulin signaling by accelerating autophagy-lysosomal degradation of LRP-1 and IR-β in blood-brain barrier endothelial cells *in vitro* and in 3XTg-AD mice. Mol Cell Neurosci.

[102] (2008). Regulation of Aβ pathology by beclin 1: a protective role for autophagy?. J Clin Invest.

[103] (2008). The autophagy-related protein beclin 1 shows reduced expression in early Alzheimer disease and regulates amyloid β accumulation in mice. J Clin Invest.

[104] (2013). Effects of membrane interaction and aggregation of amyloid β-peptide on lipid mobility and membrane domain structure. Phys Chem Chem Phys.

[105] (2015). The zinc ionophore clioquinol reverses autophagy arrest in chloroquine-treated ARPE-19 cells and in APP/mutant presenilin-1-transfected Chinese hamster ovary cells. Neurobiol Aging.

[106] (2005). P1-12 Modulation des voies mTOR, p70S6K et ERK du contrôle traductionnel par le peptide amyloïde Ab 1-42 dans des cellules de neuroblastomes humains. Rev Neurol.

[107] (2013). Aβ secretion and plaque formation depend on autophagy. Cell Rep.

[108] (2015). Autophagy is involved in oral rAAV/Aβ vaccine-induced Aβ clearance in APP/PS1 transgenic mice. Neurosci Bull.

[109] (2016). Moderate activation of autophagy regulates the intracellular calcium ion concentration and mitochondrial membrane potential in beta-amyloid-treated PC12 cells. Neurosci Lett.

[110] (2017). ErbB2 regulates autophagic flux to modulate the proteostasis of APP-CTFs in Alzheimer's disease. Proc Natl Acad Sci U S A.

[111] (2015). Impaired vascular-mediated clearance of brain amyloid beta in Alzheimer's disease: the role, regulation and restoration of LRP1. Front Aging Neurosci.

[112] (2015). Central role for PICALM in amyloid-β blood-brain barrier transcytosis and clearance. Nat Neurosci.

[113] (2018). Aβ_1-42_ oligomer induces alteration of tight junction scaffold proteins *via* RAGE-mediated autophagy in bEnd. 3 cells. Exp Cell Res.

[114] (2019). Metformin improves learning and memory in the SAMP8 mouse model of Alzheimer's disease. J Alzheimer's Dis.

[115] (2020). Activation of PPARA-mediated autophagy reduces Alzheimer disease-like pathology and cognitive decline in a murine model. Autophagy.

[116] (2013). Rapamycin attenuates the progression of tau pathology in P301S tau transgenic mice. PLoS One.

[117] (2020). A small molecule transcription factor EB activator ameliorates beta-amyloid precursor protein and Tau pathology in Alzheimer's disease models. Aging Cell.

[118] (2022). Celastrol, a TFEB (transcription factor EB) agonist, is a promising drug candidate for Alzheimer disease. Autophagy.

[119] (2020). A natural product solution to aging and aging-associated diseases. Pharmacol Ther.

[120] (2018). Metformin treatment prevents amyloid plaque deposition and memory impairment in APP/PS1 mice. Brain Behav Immun.

[121] (2010). AMP-activated protein kinase signaling activation by resveratrol modulates amyloid-β peptide metabolism. J Biol Chem.

[122] (2018). Resveratrol and Alzheimer's disease. From molecular pathophysiology to clinical trials. Exp Gerontol.

[123] (2020). Berberine in the treatment of metabolism-related chronic diseases: a drug cloud (dCloud) effect to target multifactorial disorders. Pharmacol Ther.

[124] (2006). Berberine, a natural plant product, activates AMP-activated protein kinase with beneficial metabolic effects in diabetic and insulin-resistant states. Diabetes.

[125] (2017). Berberine improves cognitive impairment by promoting autophagic clearance and inhibiting production of β-amyloid in APP/tau/PS1 mouse model of Alzheimer's disease. Exp Gerontol.

[126] (2014). Rapamycin: one drug, many effects. Cell Metab.

[127] (2010). Inhibition of mTOR by rapamycin abolishes cognitive deficits and reduces amyloid-β levels in a mouse model of Alzheimer's disease. PLoS One.

[128] (2011). Inducing autophagy by rapamycin before, but not after, the formation of plaques and tangles ameliorates cognitive deficits. PLoS One.

[129] (2019). Early intrathecal infusion of everolimus restores cognitive function and mood in a murine model of Alzheimer's disease. Exp Neurol.

[130] (2014). Temsirolimus promotes autophagic clearance of amyloid-β and provides protective effects in cellular and animal models of Alzheimer's disease. Pharmacol Res.

[131] (2011). TFEB links autophagy to lysosomal biogenesis. Science.

[132] (2015). Neuronal-targeted TFEB accelerates Lysosomal degradation of APP, reducing Aβ generation and Amyloid plaque pathogenesis. J Neurosci.

[133] (2016). The Autophagy-Lysosomal pathway in Neurodegeneration: a TFEB perspective. Trends Neurosci.

[134] (2016). A novel curcumin analog binds to and activates TFEB in vitro and in vivo independent of MTOR inhibition. Autophagy.

[135] (2016). Protein kinase C controls lysosome biogenesis independently of mTORC1. Nat Cell Biol.

[136] (2017). Effects of the insulin sensitizer Metformin in Alzheimer disease: pilot data from a randomized placebo-controlled crossover study. Alzheimer Dis Assoc Disord.

[137] (2016). Metformin in Amnestic mild cognitive impairment: results of a pilot randomized placebo controlled clinical trial. J Alzheimer's Dis.

[138] (2019). Clinical and biological effects of long-term lithium treatment in older adults with amnestic mild cognitive impairment: randomised clinical trial. Br J Psychiatry.

[139] (2022). Low dose lithium treatment of Behavioral complications in Alzheimer's disease: Lit-AD randomized clinical trial. Am J Geriatr Psychiatry.

[140] (2018). Lithium treatment for agitation in Alzheimer's disease (Lit-AD): clinical rationale and study design. Contemp Clin Trials.

[141] (2013). Latrepirdine improves cognition and arrests progression of neuropathology in an Alzheimer's mouse model. Mol Psychiatry.

[142] (2016). Metformin facilitates amyloid-β generation by β- and γ-Secretases *via* Autophagy activation. J Alzheimer's Dis.

[143] (2013). Trehalose rescues Alzheimer's disease phenotypes in APP/PS1 transgenic mice. J Pharmacy Pharmacol.

[144] (2015). Nilotinib and bosutinib modulate pre-plaque alterations of blood immune markers and neuro-inflammation in Alzheimer's disease models. Neuroscience.

[145] (2020). Berberine mitigates cognitive decline in an Alzheimer's disease mouse model by targeting both tau hyperphosphorylation and autophagic clearance. Biomed Pharmacother.

[146] (2013). Over-expression of heat shock factor 1 phenocopies the effect of chronic inhibition of TOR by rapamycin and is sufficient to ameliorate Alzheimer's-like deficits in mice modeling the disease. J Neurochem.

[147] (2010). Molecular interplay between mammalian target of rapamycin (mTOR), amyloid-β, and Tau: effects on cognitive impairments. J Biol Chem.

[148] (2015). Rapamycin ester analog CCI-779/Temsirolimus alleviates tau pathology and improves motor deficit in mutant tau transgenic mice. J Alzheimer's Dis.

[149] (2013). Autophagy enhancer carbamazepine alleviates memory deficits and cerebral amyloid-β pathology in a mouse model of Alzheimer's disease. Curr Alzheimer Res.

[150] (2016). Rifampicin is a candidate preventive medicine against amyloid-β and tau oligomers. Brain.

[151] (2018). Aspirin induces Lysosomal biogenesis and attenuates Amyloid plaque pathology in a mouse model of Alzheimer's disease *via* PPARα. J Neurosci.

[152] (2019). Cinnamic acid activates PPARα to stimulate Lysosomal biogenesis and lower Amyloid plaque pathology in an Alzheimer's disease mouse model. Neurobiol Dis.

[153] (2016). Gypenoside XVII enhances Lysosome biogenesis and Autophagy flux and accelerates Autophagic clearance of Amyloid-β through TFEB activation. J Alzheimer's Dis.

[154] (2011). Long-term treatment with lithium alleviates memory deficits and reduces amyloid-β production in an aged Alzheimer's disease transgenic mouse model. J Alzheimer's Dis.

[b155] 155ClinicalTrials.gov. Effect of insulin sensitizer metformin on ad biomarkers[EB/OL]. [2022-12-17]. https://clinicaltrials.gov/ct2/show/NCT01965756.

[b156] 156ClinicalTrials.gov. Metformin in Alzheimer's dementia prevention (MAP)[EB/OL]. [2022-12-17]. https://clinicaltrials.gov/ct2/show/NCT04098666.

[b157] 157ClinicalTrials.gov. Rapamycin–Effects on Alzheimer's and cognitive health (REACH)[EB/OL]. [2022-12-17]. https://clinicaltrials.gov/ct2/show/NCT04629495.

[b158] 158ClinicalTrials.gov. Cognition, Age, and RaPamycin Effectiveness-DownregulatIon of the mTOR-pathway (CARPE DIEM)[EB/OL]. [2022-12-17]. https://clinicaltrials.gov/ct2/show/NCT04200911.

[159] (2014). Long-term, low-dose lithium treatment does not impair renal function in the elderly: a 2-year randomized, placebo-controlled trial followed by single-blind extension. J Clin Psychiatry.

[160] (2011). Disease-modifying properties of long-term lithium treatment for amnestic mild cognitive impairment: randomised controlled trial. Br J Psychiatry.

[b161] 161ClinicalTrials.gov. Lithium as a treatment to prevent impairment of cognition in elders (LATTICE) [EB/OL]. [2022-12-17]. https://clinicaltrials.gov/ct2/show/NCT03185208.

[b162] 162ClinicalTrials.gov. Effect of lithium and divalproex in Alzheimer's disease[EB/OL]. [2022-12-17]. https://clinicaltrials.gov/ct2/show/NCT00088387.

[b163] 163ClinicalTrials.gov. A phase 3 efficacy study of dimebon in patients with moderate to severe Alzheimer's disease[EB/OL]. [2022-12-17]. https://clinicaltrials.gov/ct2/show/NCT00912288.

[164] (2008). Effect of dimebon on cognition, activities of daily living, behaviour, and global function in patients with mild-to-moderate Alzheimer's disease: a randomised, double-blind, placebo-controlled study. Lancet.

[b165] 165ClinicalTrials.gov. A Phase 3 study to evaluate the safety and tolerability of dimebon patients with mild to moderate Alzheimer's disease[EB/OL]. [2022-12-17]. https://clinicaltrials.gov/ct2/show/NCT00838110.

[166] (2018). A randomized, double-blind, placebo-controlled trial of resveratrol with glucose and malate (RGM) to slow the progression of Alzheimer's disease: A pilot study. Alzheimers Dement (N Y).

[167] (2017). Resveratrol regulates neuro-inflammation and induces adaptive immunity in Alzheimer's disease. J Neuroinflammation.

[168] (2021). Research Attitudes Questionnaire scores predict Alzheimer's disease clinical trial dropout. Clin trials.

[169] (2016). Nilotinib effects in Parkinson's disease and Dementia with Lewy bodies. J Parkinsons Dis.

[b170] 170ClinicalTrials.gov. Mycose administration for healing Alzheimer Neuropathy (MASHIANE) (MASHIANE)[EB/OL]. [2022-12-17]. https://clinicaltrials.gov/ct2/show/NCT04663854.

